# Challenges of the Medical Ethics PhD curriculum in Iran: A qualitative study

**DOI:** 10.22088/cjim.13.3.498

**Published:** 2022

**Authors:** Fatemeh Khansari, Fariba Asghari, Sara Mortaz Hejri, Fatane Bathaie, Bagher Larijani

**Affiliations:** 1 *Medical Ethics and History of Medicine Research Center, Tehran University of Medical Sciences, Tehran, Iran.*; 2 *Department of Medical Education, Tehran University of Medical Sciences, Tehran, Iran*; 3 *Faculty of Medicine, Tehran University of Medical Sciences, Tehran, Iran*; 4 *Endocrinology and Metabolism Research Center, Endocrinology and Metabolism Clinical Sciences Institute, Tehran University of Medical Sciences, Tehran, Iran*

**Keywords:** Ethics, Medical, Education, Curriculum

## Abstract

**Background:**

Bioethics is the foundation of medical practices, and can be applied in the different levels of medicine. In Iran, Medical Ethics started to be taught in the form of PhD course in Tehran University of Medical Sciences (TUMS) in 2007. Although many aspects of this plan are successfully implemented, some deficits also are frequently pointed out by many professors and students. The purpose of this study was to recognize the deficits and weaknesses of the current curriculum.

**Methods:**

This study was a qualitative descriptive type which was conducted based on semi-structured deep interview with open questions. The sample population of this research was composed of faculty members in Medical Ethics department of TUMS, students and graduates of PhD in Medical Ethics and also, the experts who worked on codification of the initial curriculum.

**Results:**

Overall, eleven individuals were interviewed. In general, “Practical application”, “Feeling the Need”, “Professional Doctorate”, “Human Sciences”, “Paramedical”, “Possible”, “Impossible”, “Defining the Discipline Nature”, “Student Attraction”, “Professor”, “Training”, “Evaluation Procedure”, “Student Admission”, “Educational Content”, “Teaching Method”, :Student Evaluation”, and “Course Management” were the main themes.

**Conclusion:**

With regard to the deficiencies in Medical Ethics training and also, the problems identified through interviews, it seems that a great deal of problems are possible to solve if Medical Ethics is considered an interdisciplinary field instead a monodisciplinary one. One of the main purposes in interdisciplinary fields is investigating, analyzing and introducing measures for issues and problems that cannot be known and solved by a single discipline.

Since bioethics is the foundation of medical practices, it can be applied in different levels of medicine. Three extant areas and fields are defined and explained for medical ethics. The first field relates to scientific and academic facet of ethics which relies on theoretical scientific aspects; i.e. how specific medical obligations and responsibilities influence ethical regulations and recommendations and vice versa, and what are values, anti-values, goodness, badness, trueness, fault, should, should not and obligations in medicine and how we can observe these considerations in practice (1). The second field relates to general policies and legal bioethics and it scrutinizes the considerations that refer to how and to the extent the legal and meta-legal institutions and organizations participate in clinical and research regulations and commitments. The third field is linked to clinical ethics and it directly focuses on how to improve the quality of patient care under the influence of medical ethics and clinical work (2).

Indeed, Medical Ethics, a multidisciplinary and interdisciplinary field, links these three fields to each other and it makes clinical physicians and scientists of other fields sit around a same table. Medical ethics is interconnected with moral philosophy, humanitarian and social rights and also, religious regulations and terms governing a society (3). In addition to law, this discipline in Iran is also interwoven with some jurisprudence issues. According to Islamic-Iranian approach, it seems necessary to localize this discipline to become applicable. Although it might be claimed that medical ethics is historically as old as medicine, only three decades have passed from its emergence in academic space.

In Iran, we face lack of trained and educated forces in this field; therefore, for the first time in 2004, Medical Ethics started to be taught in the form of MPH course in Tehran University of Medical Sciences, focusing on morality in medicine (4). The purpose of teaching Medical Ethics was to ethically organize the health-care system and train qualified forces. The main approach, also, was based on resolving the ethical challenges and problems. PhD courses in Medical Ethics are accepted in various scientific associations of the world and they are taught in different credible universities. Moreover, the need to academically train qualified forces has been recognized. Therefore, after several courses were held, the authorities decided to establish PhD in Medical Ethics. Then, different outstanding and prominent professors were invited to participate and Planning Committee of PhD in Medical Ethics was established in 2007 (4). Finally, in the 37^th^ session of Supreme Council of Planning for Medical Sciences held by the Ministry of Health and Medical Education (MOHME) in 2008, the plan was ratified and approved (4). The first PhD students were admitted in Tehran, Shahid Beheshti and Shiraz Universities of Medical Sciences in January-February, 2009, after National Entrance Exam and interviews. 

Now, it takes 4 years to graduate from Medical Ethics and the educational system of this discipline corresponds to PhD courses policies ratified by Supreme Council of Planning for Medical Sciences. In the current curriculum,a total course considered are 42 units that are theoretical and practical. Among these 42 units, 18 are compulsory specified; 4 are optional specified (according to dissertation topic); and 20 are dedicated to the final dissertation. Moreover, the students are required to pass 16 units of compensatory lessons, according to the verdict of medical department (5) .Although many aspects of this plan are successfully implemented, some deficits and weaknesses also are identified that are frequently pointed out by many professors and students (6). Some of the courses included as Medical Ethics in the curriculum have failed to help increase physicians' ethical skills (7). Efforts to strengthen the principles of Medical Ethics should be in accordance with the needs, expectations of the target groups, and social orientations (8). The Ph.D. program of Medical Ethics is a nascent course in our country and its curriculum is more modelled than the Medical Ethics curriculum of other countries, which has been designed and localized by our country's experts. Therefore, there are many questions about the comprehensiveness and quality of the elements of this program. 

On the other hand, due to the continuous and accelerating changes in medical science and technology, new issues and challenges are raised in the field of medical ethics and related issues. As a result, failure to update and revise the Medical Ethics curriculum will eventually make it an obsolete curriculum. Obviously, the implementation of such a program without a deep revision, in addition to the fact that it can lead to the waste of human and financial resources, will not meet the ultimate goal of this curriculum, namely the training of efficient specialists in the field of medical ethics (6). Hence, it is more than ever necessary to review and criticize different dimensions and details of the current curriculum based on the obtained experiences, and also, to find the gaps and deficiencies of the plan. The purpose of this study was to recognize, analyze, and explain the deficiencies and weaknesses of the current curriculum and the main root of problems in Medical Ethics PhD.

## Methods

This study is of qualitative descriptive type which is conducted based on interview method. To collect the required data, semi-structured deep interviews with open questions were performed. The statistical population of this research was composed of Faculty members in Medical Ethics department of Tehran University of Medical Sciences, students and graduates of PhD in Medical Ethics and also, the experts who worked on codification of the initial curriculum. The interviewees were selected from the statistical population using purposeful sampling method. The criteria for selecting the samples include expertise, experiment and knowledge of the individuals about the research topic. The interview was performed in the form of a semi-structured conversation. The questions of interview were designed based on Kern’s framework for curriculum review. The 6-step Kern's framework is widely used to develop medical education curriculum across various specialties and disciplines (9). This framework has become the most suitable foundation for curriculum development due to its proven efficacy and versatility (10). The framework steps include: 1- Problem identification and general needs assessment, 2- Targeted needs assessment, 3- Goals and objectives, 4- Educational strategies, 5- Implementation, 6- Evaluating the effectiveness of the curriculum (9). 

The interviews were performed in summer and fall of 2018. The process of scripting the interviews was performed at the time of and after each interview session,hence the recorded voice of interview was scripted and written. Using deductive approach based on the Kerns’ framework, thematic content analysis of the data was done manually and the extracted concepts from each question were categorized below the very question. The concepts were manually assigned to sentences, paragraphs, or sections of text. Each concept represented a major theme. After coding the first interview, in the next interview, the concepts obtained from the same question categories were added to the previous ones and the concepts containing common elements were merged together. If clarification was needed, the researcher again referred to the descriptions in the interview texts. The review process continued through repeated reading of concepts to identify common subthemes. This way, it was easy to recognize whether or not the interviews were saturated and some concepts were repetitively stated. After interviewing, 11 individuals and extracting the codes, no new concept and code were seemed to emerge in the conversations and the interviews were saturated. For data credibility, manuscripts were reviewed and verified by participants to resolve any coding ambiguities (member check). For confirmability, by systematically collecting data and observing the impartiality of the researchers, members' agreement on interviews, codes, and classification of similar codes and classes was used to compare what the researcher perceived with what the participants intended (peer check).

Since the interviewees emphasized some important notes, a coefficient called “Emphasis Coefficient” was calculated to represent the emphasis on each concept. 

*Emphasis Coefficient = (Number of Times a Code is Referred ÷ Number of Interviewees) × 100*

In case of the lack of practical courses, for example, the participants responded to the question of “Lesson Contents” with some complaints about lack or shortage of practical lessons. They uttered sentences such as *“No internship has not been seen”* or *“Clinical Ethics and a practical course for Ethics in Research should be included in syllabus”*; moreover, they pointed to these issues, again, when talking about teaching methods and they uttered sentences such as *“We haven’t visited even some patients”*, *“We did not make any real decisions in clinics”* and *“The students should walk in hospitals and ministry”*. These issues were sometimes repeated for several times within an individual’s speech. Given that this research is a qualitative one and the number of participants is not as much as that in quantitative research (so, it is not possible to determine percentage or p-value), Emphasis Coefficient was used to display the high importance of some topics and their superiority over the others. In a similar research, Dr. Naderian has previously used Emphasis Coefficient to study the feasibility of establishment of Sport Law discipline (11).

Hundred-percent emphasis coefficient shows relative agreement among the respondents, i.e. the desired concept was mentioned as many times as the number of participants. However, if the coefficient value exceeds 100%, it means that the respondents directly mentioned the concept more than once; so, it is concluded that the concept is of great importance. This event was helpful in determining the main emphasized axes to review the curriculum of Medical Ethics. 

Throughout the interview, nine questions were asked: 

1. In your opinion, what is the purpose of admitting students in PhD in Medical Ethics? What should be the purposes ideally and practically?

2. In your opinion, what are the pros and cons of the current procedure for student admission in Medical Ethics field?

3. In your opinion, what are weaknesses and strengths of the current curriculum?

4. In your opinion, which lessons lack or considered weak in the current curriculum?

5. In your opinion, what are the pros and cons of the current teaching method?

6. In your opinion, what are weaknesses and strengths of the measurement procedures for each course and the whole 4 years? 

7. All in all, how do you criticize the current education system for PhD in Medical Ethics?

8. What suggestions do you have to improve the curriculum and to empower the graduates of this field?

9. In your opinion, to what extent can Electronic Learning of Medical Ethics be helpful for those working in health care field?

The abovementioned questions were the initial and primary ones asked to direct the interview. According to the participants’ opinions, some other questions were designed and asked to clarify the real ideas of them. The interviews were conducted without any prejudice about the accuracy of the interviewees' answers. The time and place of the interviews were chosen according to the opinion of the interviewees and previous arrangements. Each interview lasted about 45 to 60 minutes. Prior to the start of the interview process, the researcher introduced herself and explained the purpose of the study and the confidentiality of the data obtained from the participants. Informed consent form was signed by the participants before each interview. They were also assured that they could refrain from continuing the study at any stage of the study. Interviews were recorded with the verbal permission of the interviewees. The researcher tried to obtain mental information about their experiences and opinions about the objectives of the research by establishing effective communication and interaction with the interviewees and guiding them to the subject.

The study has been approved by the Ethics Committee of Tehran University of Medical Sciences with the approval ID IR.TUMS.MEDICINE.REC.1396.4115.

## Results

Overall, 11 students and faculty members were interviewed. The participants’ characteristics are provided in table 1. In general, the main themes included “Practical application”, “Feeling the Need”, “Professional Doctorate”, “Human Sciences”, “Paramedical”, “Possible”, “Impossible”, “Defining the Discipline Nature”, “Student Attraction”, “Professor”, “Training”, “Evaluation Procedure”, “Student Admission”, “Educational Content”, “Teaching Method”, “Student Evaluation”, and “Course Management”. The main themes and related sub-themes are presented in tables 2–7.

**Table 1:** Interviewees’ characteristics

**Specialty**	**Gender**	**Age, years**	**Working Experience** **, years**
Medical Ethics, PhD/ Faculty member	Male	48	15
Medical Ethics, PhD/ Faculty member	Male	38	3
Clinical Pharmacist, PharmD/ Faculty member	Female	45	5
Medical Ethics, PhD & Social Medicine Specialist, MD/ Faculty member	Male	45	5
Medical Ethics, PhD & Philosophy, PhD/ Faculty member	Male	42	3
Medical Ethics, PhD & Lawyer, PhD/ Faculty member	Male	48	10
Medical Ethics, PhD & Epidemiologist, PhD/ Faculty member	Male	45	10
Medical Ethics, PhD student/ Expert in the field	Female	35	0
Medical Ethics, PhD/ Expert in the field	Female	48	10
Medical Ethics, PhD/ Faculty member	Male	45	5
Medical Ethics, PhD student/ Expert in the field	Female	45	4

***Purpose of Designing Medical Ethics as an Academic Discipline***

The first question we asked the participants related to the purpose of designing Medical Ethics in PhD grade. One of the most important answers emphasized by many of the respondents was that: due to development of medical science and emergence of sophisticated issues in treatment and also, in education and research, the need to train students in academic and higher-education levels has been recognized. In three of the respondents’ opinions, this discipline was established simultaneously with emergence of discussions about assisted reproduction and organ transplant. These respondents, also, stated that these sophisticated issues call for not only law, but also ethics and morality. 

*“The main purpose is to train students who can improve medical ethics; but to put this knowledge into practice, they should know the modern medical ethics in the world… the* *other purpose is education, i.e. teaching theoretical* *foundations and providing protocols and guidelines for physicians”.*

The reasons and purposes stated by the respondents are presented in table 2.

**Table 2:** The purpose of establishing Medical Ethics discipline from the interviewee’s perspectives

**Theme**	**Sub-theme**	**Frequency**	**Emphasis Coefficient**
Practical application	Training	19	172%
	Providing services such as consultation	37	336%
	Islamization of the ethics	2	18%
	Improving the physician’s ethics and developing their profession	6	54.5%
	Role modeling	3	27%
	Ethical policy-making (management)	18	163%
	Research	12	109%
	Theorizing and increasing awareness	13	118%
Feeling the Need	Practicalizing the ethics	8	154%
	Developing ethical discipline	4	
	Sophistication of medicine	4	
	Need of medical society to training and education	1	

**Table 3:** Eligibility and qualification to academically study in Medical Ethics

**Theme**	**Sub-theme**	**Frequency**	**Emphasis Coefficient**
Professional doctorate	Physician	26	236%
Human Sciences	Law, Philosophy, Sociology	9	81%
Paramedical	Nursery- Midwifery	9	81%

**Criteria for student admission; and target students for Medical Ethics: **An important question was proposed when reviewing the curriculum related to the criteria for admission of students and other individuals qualified to start PhD in Medical Ethics. The first issue stated by the respondents was about the educational background of students entering the course. However, disagreement also was observed. Of the eleven interviewees, five emphasized that only the graduate of professional doctorate of medicine, dentistry and pharmacy can enter PhD in Medical Ethics. Three of the respondents believed that the MS students of Paramedical are helpful and necessary to enter the courses. And the other three participants stated that MA students of human sciences including philosophy, law, sociology and psychology should also be allowed to study Medical Ethics. However, each of the respondents proposed their own reasons. The need for the applicant to be a physician, the need to graduate from paramedical disciplines and the need to graduate from human sciences disciplines were emphasized 26, 9 and 9 times, respectively (table 3). The participants who sided the entrance of medical students argued that: “The title of this discipline is “MEDICAL ETHICS”; it is an interdisciplinary field and it has two cores: philosophy and medicine. All other are around these two cores. We should admit the students of one of these fields and teach the other field. It seems much more difficult to admit the students of philosophy and teach them medicine. It’s better to admit medical students and teach them philosophy”; or “If we seek ethical reasoning, we should look for candidates who have 5-year experience in treatment, in addition to studying medicine. I do not agree with admission of candidates who are not physician or do not work in medicine field because they will fail in practical parts. These kinds of candidate will only become theorists”. Three of the participants agreed with the entry of graduates in human sciences and paramedical discipline: “We should get help from human sciences and stop keeping to medicine. We can receive help from students of Nursing and Midwifery”.

**Procedure of Student Admission: **The next question related to the procedure of student admission. All of the participants showed a consensus about selecting and admitting the students based on written entrance exam and oral interview. They believed that the current procedure for selection and admission of the students is appropriate and desirable. However, all the interviewees suggested that the oral exam and entrance interview should be more severe and determinant. 

“The students who enter this discipline must be qualified in different aspects such as reputation in the profession, commitment to professional obligations and etc. It might be better to perform the admission procedure conversely; meaning first, the students should be orally interviewed to be measured in terms of the abovementioned parameters and then they should participate in the written exam to be assessed based on their knowledge. No veto right is considered in oral interviews and the students may reach the university with the lowest level of knowledge and literacy and even with low levels of motivations”. 

**Course Content: **Given that the questions were designed based on Kern’s framework for curriculum review, the next question was related to the course content. To avoid inducing any default thought or background opinion, the current curriculum content was not given to the interviewees. However, they mentally had some ideas about the curriculum and pointed to the gaps and deficiencies in the current contents. They believed that philosophy, moral philosophy, psychology of ethics, sociology, moral decision making and especially, practical courses and clinical ethics must be more seriously considered and studied. One of the students gave an opinion about the total curriculum content: “Medical ethics and ethics in research were desirable and good; but the committees should be intended to get help from the students. A practical course might be okay; we will both help and learn. We had a worthless discussion about Computer Data. Instead of that, they could teach some details about Endnote and Referencing to the students who were eager to write. We did not learn anything about philosophy. Sociology and psychology of ethics are also, necessary. Two course units will not turn us into a psychologist or sociologist but at least, we can learn their language. They can hold these courses in the form of workshops. Psychology of ethics is really indispensable”. Three of the interviewees stated that some course units of Law and Jurisprudence do overlap so that some of them are redundant: “Five units for law and six units for jurisprudence are heavy and redundant”, “It is enough for students to get familiar with and know about the literature of jurisprudence. 

They are going to make contact with jurisprudents; so, they should know about the value of jurisprudence and its literature. They are not going to publish jurisprudential rulings and sentences. However, law is different because the students must learn the jurisdictional regulations and procedures. The students cannot match law and ethical codes; so they should learn about it. Law should be much more considered than jurisprudence”. 

The curriculum proposed by the interviewees and the grade of emphasis on each are presented in table 4.

**Table 4:** Necessary courses and lessons of Medical Ethics from the interviewee’s perspectives

	**Course Title**	**Frequency**	**Emphasis Coefficient**
Necessary Courses	Clinical/ General Medicine Ethics	6	54.5%
	Ethics in Research	1	9%
	Philosophy in Medicine	18	163%
	Philosophy of Science	14	127%
	Philosophy of Ethics	13	118%
	Ethical Consultation	10	90.9%
	Research Method and Methodology	4	36%
	Psychology of Ethics	10	90.9%
	Authorship (Writing)/ Literature	4	36%
	Principles of Jurisprudence	4	36%
	Law	9	81.8%
	Sociology	9	81.8%
	Professional Ethics	7	63.6%
	Ethical Decision Making	4	36%
	Health Economics	2	18%
	Principles of Philosophy	4	36%
	Law Philosophy	4	36%
	Logics	6	54.5%
	International Documents	1	9%
	History of Medical Ethics	5	45%
	Practical Courses	11	100%

**Teaching Method: **The next question was related to the method and manner of providing the lessons and the efficiency of each method. Since Medical Ethics is an interdisciplinary discipline, the interviewees mentioned methods such as speaking (lecture), seminars and role modeling. However, the necessity of providing practical courses and practices such as consultation, ethical decision making and ethical assessment of research plans were again emphasized. Moreover, the interviewees stated that the students should necessarily experience working in hospitals, ethics committees and even, health policy-making organizations. Three of the interviewees believed that teaching based on a monodisciplinary approach is completely wrong. They stated that: “The problem is that teaching methods are based on a monodisciplinary approach and they are totally different from interdisciplinary fields. Interdisciplinary education must be of reverse type. The students are supposed to be trained by experiencing real medical cases. After they visit 100-200 cases, they will learn the basics and fundamentals. I mean a descriptive exposure is necessary at first and then, the theoretical lessons should come next”; “The foundations are erroneously constructed. An interdisciplinary field is categorized in basic sciences group. That is why everything, from admission procedure to teaching and assessment is wrong”. 

Since internet-based and online learning is of great importance in today’s world and it is necessary for academic education system to be updated, we asked the interviewees about electronic education, separately, in addition to the questions about teaching methods. Despite the fact that online systems are now pervasive and that different educational programs are consumed in internet- and network-based forms, the interviewees did not agree with providing a total electronic course for PhD or Master grade. One of the participants believed that electronic education is applicable as one of continuous training methods, and no more. Two of the participants stated that electronic learning is a tool to provide a raw and rudimentary awareness. Another two participants, also, believed that electronic courses are effective training tools only if a balance is considered. Overall, seven participants did not accept E-learning as an educational tool and they stated that this approach would be completely ineffective because Medical Ethics requires the students to meet their professors even in completely theoretical courses. Moreover, they believed that in electronic courses, more than real courses, feigned presence of the students is likely. Please see table 5.

*“*Electronic training is inevitable and necessary; but the whole courses cannot be relied upon E-training. When the professor asks different questions in the classroom, the students’ attentions are much more highly paid to the lesson”; “Our work is based on communication. We learn how to communicate and then, we implement the learnt points in our work fields”.

**Table 5:** The interviewee’s opinion about electronic courses in Medical Ethics

**Theme**	**Sub-theme**	**Frequency**	**Emphasis Coefficient**
Possible	In the form of continuous education	2	127%
	Merely giving awareness	5	
	As a kind of educational tool	5	
	On condition of interaction	2	
Impossible	Necessity of exposure to case in medical ethics	25	418%
	Lack of covering the medical ethics goals	16	
	Possibility of students’ fake presence in electronic courses	2	
	A method for the future, not now	1	
	Disadvantages of expansion of a specific science	2	

***Assessment Procedure: *** The next question was related to the procedure of assessment for each course and the end of a semester also, to evaluate methods for the whole PhD grade in Medical Ethics. In this stage, the pros and cons of the current assessment method, both in evaluation for each course or for the whole PhD grade, were investigated and discussed and the interviewees provided some recommendations to resolve the weaknesses. One of the most important weak points of assessment for each course was inefficiency of evaluation method. Both students and professors showed a consensus about weakness of test-taking and they believed that this method cannot display and prove the students’ capabilities, alone. 

“They take exams. An individual may get good marks in written tests but she/he cannot clearly talk or make correct decisions at the time of consultation.”; “Assessments should be both practical and theoretical and undoubtedly, a part of it should be performed in written form”. 

The next weak point in assessment procedure is lack of failure possibility (i.e. failed score) for each course and for the whole PhD grade. Even the students are not likely to fail in comprehensive examination. Most of the interviewees, especially the professors, emphasized this issue. 

“The test should be just like the promotion exams for clinical medicine students”; “Failure in comprehensive examination should become possible”. 

The third weak point identified for assessment procedure was logbook completion. Most of the interviewees put emphasis on the necessity of mid-term assessment and also, on considering the influence of students’ class activities on the final score. They, however, did not agree with using logbooks for the purpose of assessment. One of their reason was related to the nature of Medical Ethics field. The current logbooks have been designed for basic sciences. Hence, many of their topics such as clinical trials and experiments are not important in Medical Ethics. Accordingly, many of the interviewees believed in inefficiency of logbooks but they stated that it is necessary and helpful to design an appropriate logbook based on the requirements of Medical Ethics discipline. 

“Logbook is an end with no beginning. It is used to evaluate the student in a term but it will not bring any help for the student. For example, it asks the students about what she/ he has done, how many speeches she/ he had or whether or not she/ he has experienced teaching. But none of these achievements are not granted to the student by the university or logbook. How could I get an opportunity to speak? The content of logbook must be corrected. A separate logbook for Medical Ethics should be designed”. 

The final question was a comprehensive open question through which the interviewees’ critiques and suggestions were sought. A great deal of answers to this question, also, related to the previous words with an emphasis on the weaknesses that need to be more seriously reformed and corrected. 

“The way curriculum is provided does not cover the purposes”; “There are no qualified and expert professors in universities”; “The foundations are erroneously built in that an interdisciplinary field is categorized in basic sciences group”; “The first step should be goal-designing. We do not even know what skills and capabilities should we search in the students”; “The position of this discipline has not been correctly defined and it is unidentified. I seriously suggest establishing an interdisciplinary college in health-care field. There are two main deficits in health care field: Theorizing and Technology. And the reason is that both of them are interdisciplinary. If the position of this profession is clearly identified and defined, the problem with individuals’ courage and dare will be resolved both managerially and legally”. 

The codes and a collection of the interviewees’ suggestions are presented in table 6. An overview of the interviews categorized in different cases (base on Kern’s model) is written in table 7. To analyze the comments better and more quickly, the pros and cons and also, the suggestions related to each section are categorized.

**Table 6:** General suggestions of the interviewee’s regarding Medical Ethics curriculum

**Theme**	**Sub-theme**	**Frequency**	**Emphasis Coefficient**
Defining the Discipline Nature	Establishing an interdisciplinary college/ or separation of the discipline from medical basic sciences	22	200%
Student Attraction	Reviewing the entrance procedure	5	90.9%
	Giving awareness about the discipline	5	
Professor	Expert and qualified professors in universities	2	18%
Training	Increase of practical courses	1	145%
	Designing proper contents	8	
	Regularly reviewing the curriculum	5	
	Providing different branches of the discipline	2	
Evaluation Procedure	Evaluation of Project Implementation	10	445%
	Evaluation of trainings and tutorials	2	
	Evaluation of activities	9	
	Evaluation of problem-solving skill	6	
	Evaluation in the whole course, not partially	2	
	Possibility of failure	5	
	Necessity of written and oral exams	15	

**Table 7:** An overview of the interview (pros and cons, and suggestions of the interviewees)

	**Strength Points of Curriculum**			**Weak Points of Curriculum**				**Suggestions**			
**Theme**	**Sub-theme**	**Frequency**	**Emphasis Coefficient**	**Sub-theme**		**Frequency**	**Emphasis Coefficient**	**Sub-theme**		**Frequency**	**Emphasis Coefficient**
Student Admission	Written exam and interview	15	136%	Lack of structured interview		6	300%	Reviewing admission procedure		5	90.9%
				Impossibility of failure for student in interview		6		Giving awareness about the discipline		5	
	Admitting students from professional doctorate*- Table 3	26	236%	Low quality of exam		11					
				Lack of ability to introduce the discipline		10					
				Lack of admitting students from other disciplines	Human Sciences	9	163%				
					Paramedical Sciences	9					
Professor				No qualified and expert professor in universities		17	154%	Hiring expert and qualified professors in universities		2	18%
Educational Content				Lack of practical courses		17	154%	Increasing practical courses		1	145%
				Lack of essential courses		32	290%	Designing appropriate content		8	
				Over-valuing some courses		10	90.9%	Regularly reviewing the curriculum		5	
				Impossibility to choose from different courses		4	36%	Providing various branches for the discipline		2	
Teaching Method	Speech	5	118%	Lack of practical courses		33	300%	Electronic courses	Possible	14	127%
	Seminar	5		Lack of severity in course holding/ weakness in course implementation		11	100%		Impossible	46	418%
	CPC	3		Limitation of max. course units for PhD students		3	27%				
	Lack of electronic courses	46	418%	Lack of attention to students’ activities		13	118%				
Student Evaluation	Written Exam	11	100%	Impossibility of failure		11	100%	Correcting the evaluation criteria		29	263%
	Writing articles	5	45%	Inefficiency		16	145%	Failure possibility		5	45%
	Logbook	21	190%	Inefficiency of logbook		4	36%	Necessity of written and oral exams		15	136%
								Designing an appropriate logbook		17	154%
Course Management				Lack of attention to the discipline nature		8	72%	Determining the discipline nature		22	200%
				Lack of severity in implementing the curriculum		25	227%	Regularly reviewing the curriculum		5	
								Providing various branches for the discipline		2	

## Discussion

Considering the emergence of some shortcomings in the field of medical ethics and the necessity of criticizing various aspects of the existing curriculum, the present study aimed at analyzing and explaining the course shortcomings as well as the root of problems in the field.

**A. To Enter Medical Ethics Field**

A problem around training Medical Ethics, which was identified throughout the interviews and on which different ideas were proposed, was the criteria for entering the field. Regarding various proposed ideas about how to enter Medical Ethics in universities, determining and defining the real nature of the field would certainly help in this issue. If it is accepted that Medical Ethics is an interdisciplinary field, then a path to various branches of its knowledge might appear. In most of the world’s leading universities such as Duquesne University (12) and Georgetown University (13) in America, Universities of Manchester (14) and Zurich (15) in Europe, Universities of Singapore (16) and Yonsei in South Korea (17) and Hacettepe University in Turkey (18) the same procedure has been implemented; i.e. the graduates in different disciplines can enter Medical Ethics and they can graduate in different branches. Admission of students from other fields requires providing various courses and prerequisite units for them; the professional life of the graduates in the future is interconnected with health care system; and ethical decision making requires comprehensive scientific knowledge about medical sciences (19). Therefore, it might be suggested that, individuals who have graduated in medical sciences would have criteria to be admitted in this course.

**B. The Purpose of Designing Medical Ethics**

According to the results of interviews, most of the interviewees believed that one of the main purposes of medical ethics is providing ethical consultation in clinics. If we accept the interdisciplinary nature of Medical Ethics, then the extent area of clinical approach umbrella would decrease and more placed become available for other purposes such as training, research, policy making and theorizing. If Medical Ethics is considered as a monodisciplinary field and if the main columns of human sciences, i.e. philosophy and law, are ignored, then theorizing will be impossible unless the purpose of Medical Ethics diminishes into training, research and clinical consultation and to the name of the field, the word “clinical” is added to clarify the real purpose of establishing such a discipline. Nonetheless, some studies declared that clinical ethics consultation requires multidisciplinary education and specific skills, so that the consultant would be able to assist patients in decision-making on critical issues in their lives (20, 21). Gross et al. and Lawlor et al. believed that medical ethics education includes various dimensions and should not be limited to learning ethical theories. Instead, professional and ethical education priorities should address the issues that students face in clinical practice (22, 23).

In general, studies indicate a lack of consensus on the main purpose of medical ethics education. Studies show that there are two points of view in this regard: 1) medical ethics education is a tool for creating virtuous physicians, 2) medical ethics education is a tool that provides physicians with the skills of analyzing and resolving ethical dilemmas. This duality is referred to as the virtue / skill duality (24-27).

**C. Curriculum Content and Teaching Method**

If Medical Ethics is categorized under the proper group (interdisciplinary field), then half of the way to select a proper curriculum is passed. The reason is that such a categorization would guide us in determining the necessary items to teach and provide to the students. When we move away from monodisciplinary approach and enter to an interdisciplinary space, we see that neither clinical courses nor jurisprudence and law merely govern. But, human sciences and medical sciences sit around the same table and all of the related sciences such as philosophy, law, psychology and sociology have something to offer. Moreover, clinical judgment is not superior to writing and analyzing. Foundations, discussions, topics and contents of each discipline considered in educational programs and courses of interdisciplinary fields are taught by teachers and professors who approach the fields according to their professional and specialized points of view and perspectives. On the other hand, one of the main advantages and special characteristics of an interdisciplinary field is that it is possible to implement a combined course or program in it, without designing a combined plan. For example, “Art Department” has prepared and provided a course in which “Art History” is taught by a historian (28). This approach is clearly considered in curriculums of Georgetown University in America (13), Universities of Manchester (14) and Zurich in Europe (15), Universities of Singapore (16) and Yonsei in South Korea (17) and Hacettepe University in Turkey (18). In these universities, courses of Authorship Technique are provided alongside with Philosophy and Law. Depending on the curriculum content, training methods might differ for different lessons and courses. 

**D. Assessment for Each Course and the Whole Term**

Effective assessment of ethics competencies is an important aspect of medical ethics education (29). In monodisciplinary fields, students’ capability and knowledge are assessed in a single specific science. However, in interdisciplinary fields, neither the courses nor assessment procedures are not structured around a single axis. If we accept Medical Ethics as an interdisciplinary field and take a path to provide all the requirements of it to study, then the assessment procedure will evolve. Under this circumstance, written and oral exams are not going to be the only ways of evaluating the students (30, 31). If we determine correct goals based on the interdisciplinary nature of Medical Ethics, we will at the end understanding what types of capabilities and skills are expected from the students. Therefore, memory work tests and ordinary exams will not suffice for evaluating the students. "SMART" approach is an appropriate approach to link student assessment to learning objectives. Hence, teaching objectives are specific, measurable, action-oriented, reasonable, and time-bound (2). This approach is based on the idea that there is an important relationship between the goals of a program and designing the assessment strategy (32). As Savulescu et al. believe, evaluation should be 1) valid: only assess relevant ethical skills and not other aspects (such as clinical competencies); 2) reliable: ensure that the same performance is assessed uniformly, no matter who assesses it; 3) relevant to clinical practice; and 4) publicly justifiable and subject to scrutiny (33). While this method can be used to tests a particular set of skills and critical thinking, Campbell et al. stated that different assessment methods are needed to assess three different ethical skills of students: 1) essay-style method to assess their knowledge; 2) case analysis method to familiarize their competencies; 3) clinical ethics examination method to assess their behavior in clinical practice (34).

**E. Necessity of Interdisciplinary Nature**

In an article entitled “Prerequisites of Interdisciplinary Human Sciences Development”, Khorshidi states that “to gain a comprehensive knowing about multidimensional phenomena of the world requires a rational transition from monodisciplinary approaches to interdisciplinary ones” (35). A fundamental and important note that is forgotten or ignored in codifying PhD in Medical Ethics is: defining the discipline nature. The codifiers, however, are not to be blamed because medical lessons and courses have long been classified into 1) basic sciences and 2) clinical sciences. Basic sciences of medicine –i.e. Anatomy, Physiology, Biochemistry and etc. –relate to only one aspect of medicine and generally, they do not interact with other fields and disciplines. These sciences are known as monodisciplinary. In practice and research, monodisciplinary sciences focus on one objective or research question within the limited area of a specific field. In monodisciplinary sciences, subject and methodological limitations of a specific field are identified and according to the problems type and methodology, the problems and questions are solved and answered (1).

While clinical sciences refer to one aspect of science, they sometimes need professional and specific comments from other medical areas. For example, surgery field requires specific knowledge about infectious diseases, heart failure or gland disorders, in some cases. Definitely, the need to those types of knowledge would be met through consulting with experts and specialists of the required field. Therefore, there is no need to consider an interdisciplinary nature for clinical sciences. In the field of clinical sciences, medicine is a multidisciplinary course in which the experts of different fields investigate and study a common subject or problem based on their own specific and cognitive perspectives. In other words, although the experts of various scientific disciplines work on a common subject, each of them attempt to reach their own goals; and while the experts try to exchange knowledge and information, neither one of them exceeds the borderlines of their fields when reaching their goals. Hence, it can be concluded that the multiple disciplines in multidisciplinary fields go ahead in parallel and at the end, no common theorization would occur (1).

In conclusion regarding the limitations and deficiencies in Medical Ethics training and also, the problems identified through interviewing the experts, it seems that a great deal of problems are possible to solve if Medical Ethics is considered an interdisciplinary field instead a monodisciplinary one. One of the main purposes in interdisciplinary fields is investigating, analyzing and introducing measures for issues and problems that cannot be known or solved by a single discipline. According to what has been stated so far, it seems that the first step to make a fundamental change in Medical Ethics curriculum is the transition from monodisciplinary nature of Medical Ethics to an interdisciplinary nature; consequently, the requirements of an interdisciplinary field have to be met for this science. Developing interdisciplinary sciences leads to different disciplines learn from each other and it causes an increase in analyzing capabilities, methodological thinking, criticizing the assumptions and a deeper insight into problems and issues of the universe. In addition to this point, more alterations seem to be necessary for instance, reviewing students’ admission process, hiring more qualified professors to teach specialized courses in the field, revising educational content of the discipline, applying more innovation in teaching methods and students’ assessment, and regularly reviewing the curriculum.
